# Functional Tea-Infused Set Yoghurt Development by Evaluation of Sensory Quality and Textural Properties

**DOI:** 10.3390/foods9121848

**Published:** 2020-12-11

**Authors:** Katarzyna Świąder, Anna Florowska, Zuzanna Konisiewicz, Yen-Po Chen

**Affiliations:** 1 Department of Functional and Organic Food, Institute of Human Nutrition Sciences, Warsaw University of Life Sciences (SGGW–WULS), 159C Nowoursynowska Street, 02-776 Warsaw, Poland; zuzanna.konisiewicz@gmail.com; 2 Department of Food Technology and Assessment, Institute of Food Science, Warsaw University of Life Sciences (SGGW–WULS), 159C Nowoursynowska Street, 02-776 Warsaw, Poland; anna_florowska@sggw.edu.pl; 3 Department of Animal Science, The iEGG and Animal Biotechnology Research Center, National Chung Hsing University, Taichung 40227, Taiwan; chenyp@dragon.nchu.edu.tw

**Keywords:** functional food, yoghurt, tea, oolong tea, sensory quality, texture properties, food design, sensory profile, consumer test

## Abstract

In the present study, the potential to design natural tea-infused set yoghurt was investigated. Three types of tea (*Camellia sinensis*): black, green and oolong tea as well as lemon balm (*Melissa officinalis* L.) were used to produce set yoghurt. The sensory quality (using Quantitative Descriptive Profile analysis and consumer hedonic test) and texture analysis, yield stress, physical stability and colour analysis were assessed to describe the profile of the yoghurt and influence of quality attributes of the product on the consumer acceptability of infused yoghurts in comparison with plain yoghurt. Among the analyzed plant additives for yoghurt, addition of 2% oolong tea to the yoghurt allows a functional food to be obtained with satisfactory texture and sensory properties, accepted by consumers at the same level as for control yoghurt. Both types of yoghurt were also characterised by high consumer willingness to buy, which confirms the legitimacy of using oolong tea as a natural, functional yoghurt additive that improves the sensory quality of the product. The high overall quality of yoghurt with oolong tea in comparison to other plant extracts was associated with the intensive peach flavour and odour, nectar and sweet odour and flavour, and the highest creaminess and thickness. That was confirmed by principal component analysis (PCA) where the overall sensory quality of yoghurts was mainly positively correlated with peach flavour and odour, sweet odour and yoghurt odour, while it was negatively correlated with herbs flavor and odour, and green tea flavour and odour. The sensory profile confirmed no differences in textural profile between plain yoghurt and the tea-infused one measured in the mouth, which corresponds to the result of textural properties such as firmness and adhesiveness.

## 1. Introduction

Functional food is defined as food that is fortified, enhanced, enriched or as a whole food that has a documented health effect beyond that resulting from the presence of nutrients traditionally considered essential [1,2]. Yoghurt, as a nutrient-rich dairy product, can be classified as functional food [3]. The main factors responsible for the beneficial effects of yoghurt are live cultures (*Streptococcus thermophilus* and *Lactobacillus bulgaricus*), proteins (whey and casein), lipids (bioactive fatty acids), vitamins and minerals (calcium and vitamin D) [4,5]. Of all the fermented products, yoghurt is also the most popular in the world [3,6] and best perceived and accepted by consumers [3,7]. Consumers who purchase functional products want the product to be safe, healthy, and natural and to have pleasant taste. They take into consideration such quality-related attributes on the labeling as freshness of the product, healthful properties, and nutritional value [8].

Dairy products belong to the most innovative food sector in Europe. The innovation of these products is based on product improvement, new formulations or new technologies that are used to meet the needs of specific consumers. Based on the research undertaken on French consumers, nutrient fortifications coming from plant sources were the most acceptable for them [9]. Several studies were conducted on yoghurts to know how yoghurt fortification with vitamins such as vitamins C, B9, B12, A and D [10] or minerals such as chromium, iron, magnesium, manganese, molybdenum, selenium and zinc [11] influenced their properties. Apart from yoghurt’s fortification with vitamins and minerals, it has become more popular to add plant-based functional ingredients to the yoghurt like pomegranate juice powder (1–5%) [12], dried pomegranate seeds (5–20%) [13], freeze-dried apple pomace powder (1–3%) [14], flaxseed (0–4%) [15], coconut-cake (0–30%) [16], spirulina (0.25–1%) [17], aloe vera gel (1–5%) [18], saffron (0.0125%) [19] or tea [20,21,22,23,24,25] to improve their technological and sensory quality as well as health-promoting properties.

Tea is the most common functional beverage in the world [1,26] usually prepared by infusing leaves of the plant *Camellia sinensis* (L.) in hot water [27,28]. Tea can be classified according to the degree of fermentation into un-fermented green tea, semi-fermented oolong tea, and fully fermented black tea [1,27,28]. Thanks to the content of polyphenols, especially epigallocatechin-3-gallate, theaflavins and thearubigins, tea from *Camelia sinensis* (L.) provides several health-promoting effects [1,26,29]. Infusions of herbs, fruits, roots and flowers are also referred to as tea, and their health benefits are known and used by people around the world [30]. One of them is *Melissa officinalis*, called lemon balm, known for its many therapeutic properties such as antioxidant, antidepressant, anti-inflammatory, and antimicrobial activities. Lemon balm can be used for both prevention and treatment in medicine as well as in dietary supplements and functional food [31]. The addition of various extracts of black, green and white tea enhanced antioxidant properties of yoghurts [23]. The influence of tea on lactic bacteria during yoghurt fermentation was also verified and it was shown that this addition did not interfere with the fermentation process and did not affect the survival of bacteria. It was also shown that lactic acid bacteria present in yoghurt did not have a negative effect on the content of tea pro-health compounds [22]. In most of the available publications, the purpose of using tea addition to yoghurt was health promotion and enrichment of these products with antioxidants and ingredients that had a positive effect on human health [20,22,23,24,32]. The sensory quality of yoghurts developed with teas has been evaluated so far by researchers using only consumer tests [15,21,25,33]. However, there is a lack of information on the influence of tea on the sensory quality of yoghurt measured both by expert panels and consumers and supported by yoghurt textural properties that are very valuable during the new product development process.

The aim of developing the new product was to maintain the functional character of nutri-rich yoghurt and, in addition, to introduce a health-promoting plant material into natural yoghurt without added sugar. That is the reason why the aim of the research was to develop functional tea-infused yoghurts by assessing the sensory quality and texture properties of yoghurts.

## 2. Materials and Methods

### 2.1. Materials

The material for the actual tests were yoghurts prepared from microfiltered pasteurised cow’s milk with 3.2% fat content (Piątnica, Poland), using the thermostatic method. Four types of leafy tea (*Camellia sinensis*) available on the Polish market were used for the production of yoghurt, i.e., green tea (BioFix, Tuszyn, Poland); black tea, Darjeeling FTGFOP1 Blend Lucky Hill (Tea Club Marek Brzezicki, Lubin, Poland) and oolong tea Oolong Milky (Herbaty Szlachetne Sp. Z o. o., Szczecin, Poland) and also the lemon balm (*Melissa officinalis* L., Cesarska Perła, Warszawa, Poland). In order to inoculate the milk, freeze-dried starter culture YO-122 (Serowar, Szczecin, Poland) containing *Streptococcus salivarius* subsp. *thermophilus* and *Lactobacillus delbrueckii* subsp. *bulgaricus* were used.

#### Yoghurt Processing

The technological process of set yoghurt production was developed based on modifications of two methods [22,23]. All ingredients were weighed on an analytical balance (RADWAG PS 1000/C/2, Radom, Poland). The milk was heated to 85 °C for 30 min. and poured into the beakers with tea leaves (2 g tea/100 mL of milk). It was steeped under a lid for 10 min, from time to time being stirred. Then the solution was manually filtered using gauze filters and cooled to 43 °C. We added 0.1% of starter cultures to milk and stirred thoroughly. Then 100 mL of milk was poured into sterile plastic containers with lids. All samples were thermostated at 43 °C in the incubator set (Memmert INE 500, Schwabach, Germany) until they reached the pH value of 4.5–4.6 (Voltcraft PH-100ATC, Wollerau, Switzerland), which took approximately 4.5 h. The samples were then removed and allowed to cool. The samples were stored at 4 °C for 15 h till the structure was built and then the sensory evaluation and instrumental analysis were undertaken [34,35,36]. Plain yoghurt was prepared similarly, only tea was not added to it.

### 2.2. Methods

#### 2.2.1. Sensory Analysis

##### Expert Test

- **The Method:** The sensory characteristics of the yoghurts were assessed using the Quantitative Descriptive Profile (QDP). A method following the procedure was described in ISO standard 13299:2016 [37]. According to the procedure, the panellists first individually chose the descriptors (attributes) of appearance, odour, consistency and flavour/taste of samples. Then the attributes were discussed, agreed, and defined by the panellists. The final list of 39 attributes with definitions is presented in Supplementary Materials (Table S1). There were 11 characteristics describing the odour of samples (milky, yoghurt, sour, sweet, fat, green tea, black tea, herbs, peach, citrus, nectar), seven describing the appearance of the samples (whey, shine, colour, smoothness visually, adhesiveness, teaspoon filling, consistency uniformity), seven attributes describing the texture felt in the mouth (thickness, melting, firmness, yield stress, fat film, creaminess, smoothness), four describing the taste (sweet, sour, bitter and astringent), eight describing the flavour (milky, yoghurt, quark, green tea, black tea, herbs, peach) and characteristics describing the body and overall quality. The intensity of each attribute was measured by panellist on a linear unstructured 10-point scale (c.u.—contractual units) where 0 means low perception while 10—high perception.
- **The Expert Panel:** Quantitative Descriptive Profile analysis of yoghurt samples was performed by 10 trained panellists (experts), women aged between 35 and 52 with a good knowledge of all of the sensory methods, including profiling and yoghurt analysis. The panellists fulfilled the requirements of ISO standard 8586:2012 [38].
- **Testing Conditions:** Sensory evaluation was performed in the sensory laboratory fulfilling all the requirements of ISO standard 8589:2007 [39]. The assessment was carried out in individual testing booths with controlled lighting, temperature, and humidity. The booths were equipped with a computerised system, ANALSENS, for experiment planning, data acquisition and processing. The assessments were conducted during the morning and early afternoon, with two sessions per day.
- **Sample Preparation and Presentation:** Five types of yoghurt (C—Control, G—Green tea, B—Black tea, O—Oolong tea, M—Lemon balm) were assessed directly from test containers. The samples were prepared in cylindrical containers (ø 50 mm, height 50 mm, volume 100 mL), coded with 3-digit codes, placed randomly on the tray, and served at 7 °C to the evaluators. Still mineral water was used as neutraliser between samples. Each sample was analyzed in two independent replications, and so the mean values were based on 20 individual results which were used for statistical analysis.

##### Semi-Consumer Test

The semi-consumer study was conducted at the Institute of Human Nutrition of Warsaw University of Life Sciences (WULS-SGGW) among students from the Faculty of Human Nutrition aged 19–31 recruited on the basis of willingness and interest to participate in the test (*n* = 30), they were randomly recruited. Thirty regular consumers of yoghurt or fermented milk products with no allergy reaction to milk participated in the study. The evaluation was carried out for five types of yoghurt: yoghurt with green tea (G), black tea (B), oolong tea (O), yogurt with lemon balm (M) and plain yoghurt as a control (C). The samples were prepared in cylindrical containers (ø 50 mm, height 50 mm, volume 100 mL), coded with 3-digit codes, placed randomly on the tray, and served at 7 °C to the evaluators. The consumers assessed the acceptability of appearance of yoghurt, their odour, taste, consistency and overall acceptability as well as willingness to buy yoghurt using a structured 9-point hedonic scale, where 1 meant “dislike extremely/will not buy definitely”, and 9 “like extremely/will buy definitely” [40,41].

#### 2.2.2. Instrumental Analysis

##### Textural Properties

Texture analysis of yoghurts was determined using a texture analyzer (TA.XT Plus, Stable Micro Mixtures, Surrey, UK) with a 5 kg load cell at 20 °C. The firmness (N) and adhesiveness (Ns) were analyzed by a 0.5 cm diameter cylindrical flat probe (P/0.5R). The measuring speed was 1.0 mm/s and the trigger force was 1 g. Samples were prepared in cylindrical containers (ø 50 mm, height 50 mm, volume 100 mL) and the penetration depth of the yoghurt was 5 mm. The reported values represented the averages of three replicates. The data were analyzed using the Exponent v6.1.4.0 equipment software [34,42].

##### Yield Stress

To measure the yield stress of yoghurts a rheometer (DV3T, Brookfield, Middleboro, MA, USA) was used. Measurements were conducted at 20 °C using spindles dedicated to yield stress (Pa) analysis: vane spindle V74 with a torque range HA. Samples were prepared in cylindrical containers (ø 50 mm, height 50 mm, 100 mL volume) and measurement was performed by controlling shear rate from 0.01–100 s^−1^. The reported values represented the averages of three replicates. The data were analyzed using the software provided with the rheometer [34,43].

##### Physical Stability—CSA Method

The changes in the yogurt stability were investigated using space- and time-resolved extinction profiles (STEP) technology. This is a new technique employing gravitational fields to accelerate the occurrence of instability phenomena such as sedimentation, flocculation or creaming [43]. The physical stability of yoghurts was determined with an analytical centrifuge LUMiSizer 6120-75 (L.U.M. GmbH, Berlin, Germany) by measuring the intensity of transmitted near-infrared light in suspension, and recording of light intensity profiles as a function of time and position of the sample (“fingerprints”) [44,45]. Stability was shown as a space- and time-related transmission profile over the sample length. The parameters used for the analysis were: wavelength 870 nm, volume 1.8 mL of dispersion; light factor: 1; 4000 rpm; experiment time, 50 min; interval time 10 s; temperature 25 °C. The reported values represented the average of six replicates. The data were analyzed by the delivered software (SepView 6.0; LUM, Berlin, Germany) and the instability index was calculated [45].

##### Colour Parameters

To determine the colour components (L*, a*, and b*) the Minolta CR-200 colorimeter (Minolta, Japan; light source D65, observer 2°, a measuring head hole of 8 mm) was used. Colour parameters were analysed using the CIEL*a*b* system. The measurements were made at the surface of yoghurts. To determine the colour differences between plain yoghurt and infused yoghurts, the parameter of total colour difference ΔE was calculated [46].

$$\Delta E=\sqrt{{\left(L_{c}^{*}-L_{T}^{*}\right)}^{2}+{\left(a_{c}^{*}-a_{T}^{*}\right)}^{2}+{\left(b_{c}^{*}-b_{T}^{*}\right)}^{2}}$$

where: $L_{c}^{*},\;a_{c}^{*},\;b_{c}^{*}$ refers to the colour parameters of plain yoghurts and $L_{T}^{*},\;a_{T}^{*},\;b_{T}^{*}$ refers to the colour parameters of tea infused yoghurts.

#### 2.2.3. Statistical Analysis

The results of texture, yield stress, stability and colour were statistically analyzed using Statistica 13.3 (TIBICO Software Inc.). To determine the significance of differences between the average values of analyzed parameters of tea-infused yoghurts, one-way analysis of variance (ANOVA) was used. Significant differences between infused yoghurts and plain yoghurt were verified using Tukey’s test at significant level α = 0.05. The results of sensory analysis were statistically analyzed using Statgraphics Plus 5.1 (Statgraphics Technologies, Inc., Plains, VA, USA). The one-way ANOVA at the significance level (*p* ≤ 0.05) was used to check the significance of differences in attributes intensity among analyzed samples. Mean values marked with different indices a, b, c, d differed statistically (*p* ≤ 0.05). Principal component analysis (PCA) was used to analyze differences between samples and correlation of selected variables. The ANALSENS NT program was used for the PCA analysis.

## 3. Results and Discussion

### 3.1. Sensory Analysis

#### 3.1.1. Preliminary Test

In order to determine the appropriate concentration of the applied tea, preliminary tests were conducted based on the evaluation of the sensory properties of the obtained tea-infused yoghurts. A preliminary study was carried out on green tea yoghurt with the concentration being 1%; 2%; 4%; 6%; 8% for individual samples respectively. After sensory evaluation, it was found that the addition of 2% green tea was sufficient and provided the best sensory characteristics, compared to the others. The higher percentage of tea in yoghurt resulted in deterioration of sensory quality, taste, odour and colour. An improvement in consistency was observed in the 6% and 8% green tea-infused yoghurts, but the intense bitterness, astringency and aroma of these samples resulted in their very low sensory quality. Based on these results, tea with different degree of fermentation (green tea, oolong tea and black tea) and lemon balm (2%) with health-promoting properties was selected for further design studies on the composition of yoghurt.

#### 3.1.2. Quantitative Descriptive Profile Analysis of Tea-Infused Yoghurts in Comparison with Plain Yoghurt (Expert Test)

The sensory quality of developed yoghurts with teas has been evaluated so far by researchers using hedonic methods [15,21,25,33] using a 9 or 7 degree scale, which has enabled them to obtain information on the acceptability of the product, but has not given full information on the sensory profile of the product, which could be obtained by using expert panel research, e.g., Quantitative Descriptive Profile analysis.

In the present study, the sensory profile was assessed for five types of yoghurts: yoghurt with green tea (G), yoghurt with black tea (B), yoghurt with oolong tea (O), yoghurt with lemon balm (M) and plain yoghurt as a control one (C) by using Quantitative Descriptive Profile analysis. The trained panellists defined 39 main attributes with their characteristics (Supplementary Materials Table S1), which described the evaluated yogurt samples. To describe the sensory experience of different food categories, a number of lexicons was developed [47,48]. Coggins and co-authors [49] developed a sensory lexicon for plain yoghurts in the United States based on 12 commercially produced yoghurts, where a trained panel defined 61 sensory descriptors. The plain yoghurt was used in the current study and, based on it, yoghurts with tea were produced. Therefore, our expert panel, in addition to the characteristics of plain yoghurt, also defined additional features describing the characteristics of the plants used in the research for the yoghurt production (Supplementary Materials Table S1).

**Figure 1:**
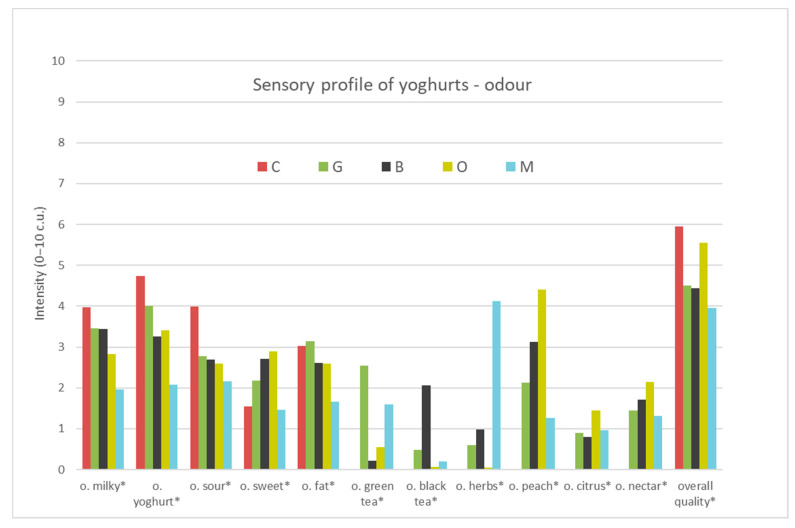
The sensory quality profile of yoghurt samples (odour). C—Control, G—Green tea, B—Black tea, O—Oolong tea, M—Lemon balm (o-odour), (*—differ significantly *p* ≤ 0.05).

**Figure 2:**
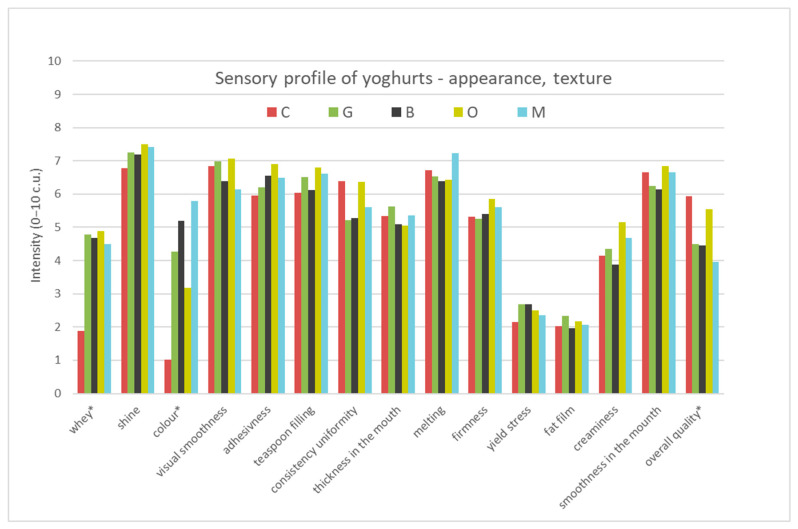
The sensory quality profile of yoghurt samples (taste and flavour) C—Control, G—Green tea, B—Black tea, O—Oolong tea, M—Lemon balm (t-taste, f-flavour), (*—differ significantly *p* ≤ 0.05).

**Figure 3:**
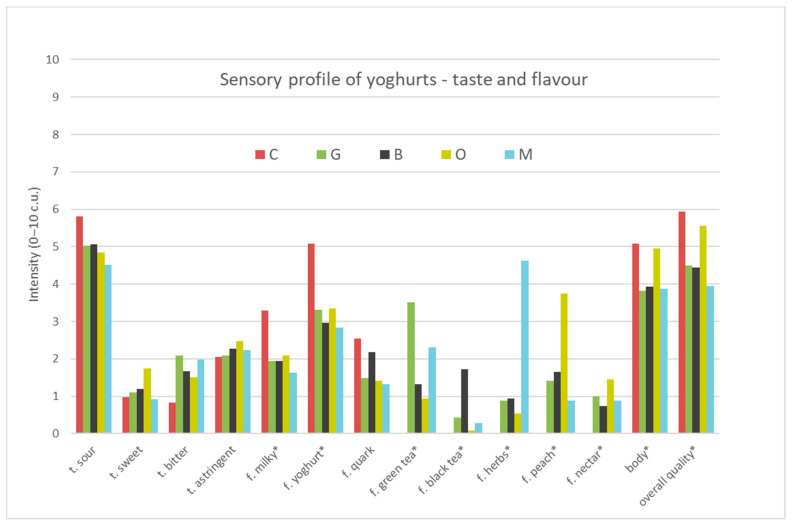
The sensory quality profile of yoghurt samples (appearance and texture). C—Control, G—Green tea, B—Black tea, O—Oolong tea, M—Lemon balm (*—differ significantly *p* ≤ 0.05).

The results of the profiling analysis of the four tea-infused yoghurts (G—Green tea, B—Black tea, O—Oolong tea, M—Lemon balm) and plain yoghurt (C—Control sample), are presented in Table S2 (Supplementary Materials) and in Figure 1, Figure 2 and Figure 3. The results showed that the samples were characterised with different sensory profiles (Figure 1, Figure 2 and Figure 3). Control yoghurt was characterised by milky, yoghurt, sour, quark profile in taste and smell, intense fatty smell, light whey flow and light creamy colour. It was also characterised by dense, creamy, uniform consistency. Green tea infused yoghurt was characterised by intensive green tea, peach and nectar odour and flavour profile and noticeable bitter and astringent taste, more intense flow of whey, darker cream colour and dense, creamy, uniform consistency. All these attributes were related to the quality of the green tea, that is characterised by yellow colour, bitter and astringent taste, as well as floral (we called it nectar), grassy or burn leaf [50]. The profile of oolong tea-infused yoghurt was characterised with the intensive peach and nectar odour and flavour, citrus odour, sweet and astringent taste, more intense flow of whey, cream colour and dense, creamy and uniform consistency. Oolong tea flavour profile depended on the time of fermentation and was described as sweet, floral, green fruity, astringent, bitter and umami [51] and most of these attributes were perceived in oolong tea-infused yoghurt. Black tea-infused yoghurt was characterised by intensive dark cream colour, uniform and dense, and creamy consistency, intense flow of whey, and intensive black tea, and less-intensive peach odour and flavour and bitter and astringent taste. Black tea that was used in the research was Darjeeling tea, Fine Tippy Golden Flowery Orange Pekoe, that was characterised with light and delicate flavour and aroma. The second flush Darjeeling tea produced excellent quality teas that were considered to be better than the first flush as they had a fruitier, less astringent flavour than the earlier teas [52]. The taste and aroma of green, black and oolong tea, depended on the degree of fermentation, and the content of free amino acids, mainly l-teanine and natural amino acids, e.g., glutamic acid and asparagine [53]. The profile of lemon balm-infused yoghurt was characterised with very intensive dark cream colour, uniform, dense and melting consistency, but also very intensive herbs and green tea odour and flavour, as well as bitter and astringent taste and citrus odour. Such a yoghurt profile might result from the addition of lemon balm, which was characterised by lemon taste and odour [31] and light yellow colour [54], but probably their composition might influence changes in the profile during the yoghurt production process.

The yoghurts developed differed significantly from each other in various attributes: odour (milky, yoghurt, sour, sweet, fat, green tea, black tea, herbs, peach, citrus, nectar), appearance (whey and colour intensity), flavours (milky, yoghurt, green tea, black tea, herbs, peach, nectar), body and overall quality. The significance of differences between compared samples in intensity of the attributes is marked in Table S2 and Figure 1, Figure 2 and Figure 3.

The detailed characteristics of smell, appearance, texture felt in the mouth, taste and flavour as well as body and overall quality of the evaluated yoghurts are described below.

##### Odour

The examined samples of yoghurt differed markedly in all odour attributes (Figure 1). The significantly highest level of milky odour was observed in the control sample (C-4.0 c.u.) (Table S2), while the lowest in yoghurt with lemon balm (M-2.0 c.u.). Also, the milky odour was less intensive in samples G, B and O than in control samples. There were no significant differences in milky odour between samples G, B and O (3.5, 3.4, 2.8 c.u., respectively). Similar dependencies were observed in the yoghurt odour, which was significantly the most intensive in the control sample (C-4.7 c.u.), while the lowest in yoghurt with lemon balm (M-2.1 c.u.). Sour odour was significantly the most noticeable in control sample (C-4.0 c.u.) while significantly least in yoghurt with green tea, black tea, oolong tea and lemon balm (G-2.8, B-2.7, O-2.6, M-2.2 c.u.). Sweet odour was statistically the least noticeable (1.5 c.u.) in the control sample and in yoghurt with lemon balm while significantly the sweetest odour was perceived in yoghurt with oolong tea (2.9 c.u.-twice more intensive than in control one). The significantly most-intensive green tea odour was in yogurt with green tea (G-2.5 c.u.), while black tea in black tea yoghurt (B-2.1 c.u.) and herb odour in yoghurt with lemon balm (M-4.1 c.u.). The oolong tea was characterised with significantly intensive peach (4.4 c.u.) odour. Citrus (0.9-1.4 c.u.) and nectar odour (1.3-2.1 c.u.) were significantly perceived in green tea, black tea, oolong tea and lemon balm. All yoghurts represented a very similar and low level of refreshing odour (2-3.2 c.u.). Tea addition to the plain yoghurt influenced the odour sensation. The smell of milk, yoghurt, sour and fatty smell was lowered, while the sweet smell was emphasised, and a typical smell of infused plants appeared.

##### Basic Taste and Flavour

The samples of yoghurts examined differed significantly in milky, yoghurt, green tea, black tea, herbs, peach and nectar flavours (Figure 2). All yoghurt samples were described as sour. The intensity of sour taste did not exceed of 5.8 c.u. on the scale in all products (Table S2). The samples showed low intensity of sweet (0.9–1.7) and bitter (0.8–2.1 c.u.) and astringent taste (2–2.5 c.u.). The significantly less bitter was control yoghurt (0.8 c.u.) and the most bitter but still on the lowest level was yoghurt with green tea (2.1 c.u.). The astringent and bitter taste was characteristic for teas due to the polyphenols present in them [26]. The examined samples of yoghurts differed significantly in flavour characteristics. The control sample had significantly more intensive milky flavor (3.3 c.u.), yoghurt flavour (5.1 c.u.) and quark flavour (2.5 c.u.) than other tea-infused yogurts, and there were no plant flavours perceived in plain yoghurt such as green tea, black tea, herbs, peach and nectar flavours. Plain yoghurt should be characterised with a pleasant, clean acid flavour, with no bitter, rancid, oxidised, yeast and unclean flavours, and be firm, with a smooth and homogeneous texture. Yoghurt taken on a spoon should keep its shape without sharp edges and should present a clean, natural white colour, with a smooth, velvety appearance [55]. The plain yoghurt tested met the above requirements, except for the bitter taste, which was perceived in a sample but not very intense. According to the literature [21], to overcome the bitter flavour of the green tea chocolate coupled with honey might be used. The product had good consumer acceptability as indicated by the sensory evaluation [21]. Green tea flavour was significantly perceived more strongly in yoghurt with green tea (3.5 c.u.) than in other plant-infused yoghurts (0–2.3 c.u.). A similar relationship occurred in the case of yoghurt with black tea where black tea flavour was significantly perceived in yoghurt with black tea (1.7 c.u.), while herb flavour was significantly perceived in yogurt with lemon balm (4.6 c.u.) than in other tea-infused yoghurts (0–0.9 c.u.). Peach flavour was significantly perceived in yoghurt with oolong tea (3.8 c.u.), less intensive in other plant yoghurts (0.9–1.7 c.u.) while the nectar flavour was significantly perceived on a similar level (0.7–1.5 c.u.) in tea yoghurts in comparison to the plain one (0 c.u.). In one study, the bitter taste of green tea was effectively masked in the yoghurt by adding chocolate and honey [21]. It was found that three types of tea extract and lemon balm affected the flavour of the plain yoghurt. The control sample was described as having a milky, yoghurt, fatty and quark flavour while in tea-infused yoghurts additional flavour of green tea, black tea, herbs, peach and nectar was perceived.

##### Visual Attributes/Appearance

While evaluating the visual attributes and appearance of the yoghurt samples, it was observed that they differed significantly only in the presence of whey and the colour of the yoghurt (Figure 3). All yoghurts represented a very similar and high level of shine (6.8–7.5 c.u.) (Table S2). The significantly lower level of whey presence was observed in control sample (1.9 c.u.), then in yoghurts with tea (4.5–4.9 c.u.). In a study analyzing the influence of green tea and moringa on the quality of yoghurt, it was found that moringa showed a greater influence on yoghurt sineresis than green tea [25]. The samples of yoghurts differed significantly in cream colour intensity. The control sample (C) was significantly lighter in cream colour (1.0 c.u.) than the rest of the samples. The yoghurt with lemon balm was characterised by significantly the darkest cream color (5.8 c.u.), as well as yoghurt with black tea (5.2 c.u.). All samples represented similar high visual smoothness (6.1–7.1 c.u.) and thickness measured by spoon resistance (6–6.9 c.u.), as well as filling the teaspoon, and was more conical (6–6.8 c.u.) than flat. All analyzed samples were uniform in consistency (5.2–6.4 c.u.). Yoghurt should have a delicate and smooth texture and a firm body, which is maintained while eating with a spoon [56]. These were the characteristics of the yoghurts developed with the addition of tea and plain yoghurt. The addition of tea resulted in significantly higher whey flow in tea-infused yoghurt than in control yoghurt, as well as its significantly darker colour, especially in yoghurt with lemon balm and black tea.

##### Texture in the Mouth

The examined samples of yoghurts did not differ significantly in texture attributes felt in the mouth (Figure 3). They were moderately medium thick (5.1–5.6), and melted very well in the mouth (6.4–7.2 c.u.) (Table S2). All yoghurt samples were characterised with medium firmness (5.3–5.8 c.u.) and low yield stress (2.1–2.7 c.u.). Fat film was perceived in all samples on the same level (2.0–2.3 c.u.). All analyzed yoghurts were characterised by moderate creaminess (3.9–5.2 c.u.) and high smoothness in the mouth (6.2–6.8 c.u.). In the case of plain yoghurt’s taste and texture, characteristics allowed trained panellists to differentiate, identify and categorise yoghurts, but it was not possible to differentiate or categorise them on the basis of percentage fat content. Natural yoghurts’ differentiation was more effective on the basis of taste and texture than aroma and appearance [49]. The addition of tea to yoghurt did not significantly affect the texture felt in the mouth. 

All the analyzed samples of yoghurts differed significantly in their bodies, a characteristic that described the harmonisation of all positive attributes. The significantly highest body had control yoghurt (5.1 c.u.) and the oolong tea one (5.0 c.u.). Yoghurts with green tea, black tea and lemon balm had significantly lower body. 

Overall quality that depended on all the characteristics perceived in the yoghurt samples was significantly different in all samples. The significantly highest overall quality had the control sample (5.9 c.u.), then yoghurt with oolong tea (5.6 c.u.), while the significantly lowest overall quality had yoghurt with lemon balm (4.0 c.u.). The biggest influence on the lowest overall quality of yoghurt with lemon balm was probably the most intensive herb flavour and odour. The high overall quality of yoghurt with oolong tea was associated with the intensive peach flavour and odour, nectar and sweet odour and flavour, and the highest creaminess and thickness. The highest overall quality of the control sample was associated with the highest yoghurt, milky and quark flavours and sour taste, which were typical for the plain yoghurt.

#### 3.1.3. Principal Component Analysis (PCA) Analysis

**Figure 4:**
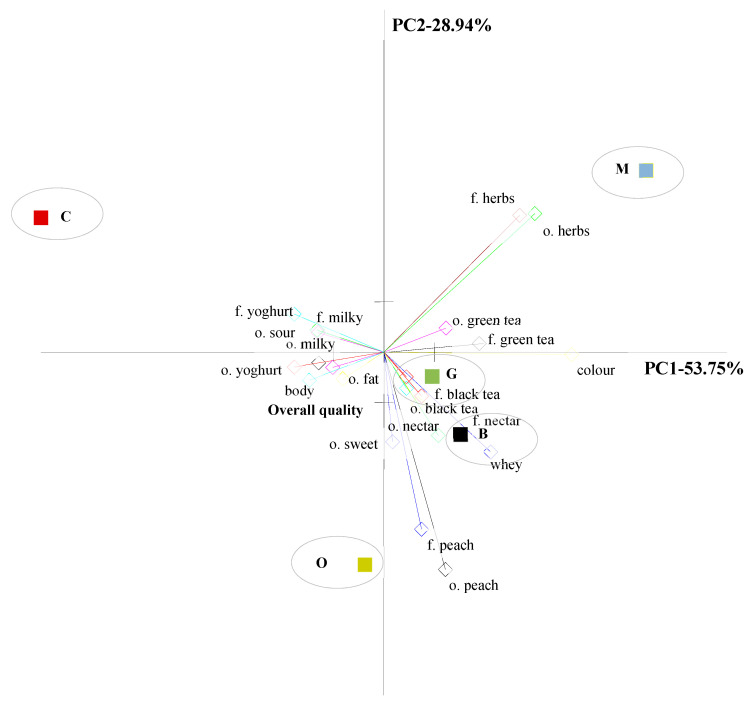
Similarities and differences in the sensory quality of yoghurt samples. C—Control, G—Green tea, B—Black tea, O—Oolong tea, M—Lemon balm (PCA) (f-flavour, o-odour).

For the evaluation of the sensory profile of 5 yoghurts, 39 attributes were defined. Analysing them all in PCA, it was possible to notice that they were all not clearly visible in the graph, so only those attributes with which the samples differed significantly (*p* ≤ 0.05) were used for PCA. The principal component analysis of the results of the profile evaluation of the all yoghurt samples showed that the variability of the samples was assigned primarily to the first main component (PC1-53.75% of the total variability) and concerned different colour, intensity of green tea flavour and odour, yoghurt, milky and sour odour, and body of the samples (Figure 4). These were the attributes that differentiated the evaluated samples. The second main component was assigned a smaller percentage of general variability (PC2−28.94%) and concerned a different intensity of peach odour and flavor, and sweet odour. Overall sensory quality of yoghurts was mainly positively correlated with peach flavour and odour, sweet odour and yoghurt odour, while it was negatively correlated with herbs flavour and odour, and green tea flavour and odour. The yoghurt samples differed in sensory quality as evidenced by their location in the space of the PCA system. The samples can be observed to form four distinctive clusters. The first one covered the plain yoghurt, the second yoghurt with lemon balm, the third yoghurt with black tea and green tea, the fourth yoghurt with oolong tea. The yoghurt with oolong tea was relatively close to the overall sensory quality in comparison to other samples and was characterised by intensive peach flavour and odour positively correlated with overall quality of yoghurt. Yogurt with lemon balm was on the opposite site of oolong tea and overall quality because of its very intensive herbal flavour and odour characteristic to that sample. Results of the PCA corresponded to the results obtained in the Quantitative Descriptive Profile analysis.

#### 3.1.4. Acceptability of Yoghurts and Willingness to Buy Evaluated by the Consumers/Semi-Consumer Evaluation of Yoghurts Based on Different Types of Tea

**Figure 5:**
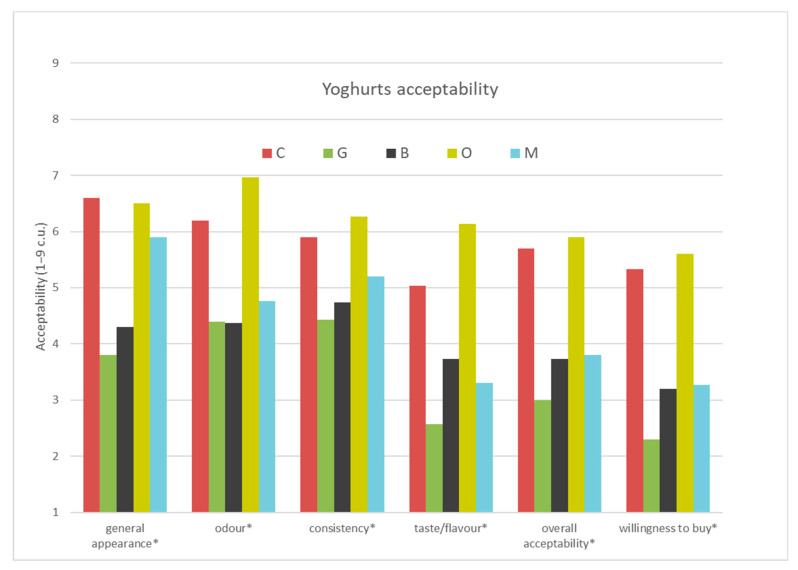
Acceptability of 5 yoghurts with tea (C—Control, G– Green tea, B—Black tea, O—Oolong tea, M—Lemon balm) and willingness to buy them (*—differ significantly *p* ≤ 0.05).

The overall goal when designing a food product is to make it sensory acceptable and desirable by consumers. In this research, apart from the expert sensory evaluation, it was decided to conduct consumer research. The semi-consumer assessment was carried out among 30 consumers, and their task was to assess the acceptability of the general appearance, odour, consistency, taste/flavour, overall acceptability of the product taking into account all the characteristics and willingness to buy four yoghurt samples differing according to the type of tea added in comparison to the plain yoghurt (Table S3). (Figure 5).

On the basis of the results obtained, it was found that the control sample (C) and the sample with the addition of oolong tea (O) (5.7 and 5.9 c.u.) (Table S3) were characterised by the significantly highest and similar overall acceptability, while the significantly least acceptable in terms of overall acceptability were samples with the addition of green tea (G), black tea (B) and lemon balm (3.0, 3.7 and 3.8 c.u. respectively). The significantly highest overall acceptability was presented in the control yoghurt and yoghurt with oolong tea which translated into a willingness to buy those yoghurts. Consumers wanted to buy yoghurt with oolong tea (5.6 c.u.) and control yoghurt (5.3 c.u.) the most, while significantly less the yoghurts with lemon balm, black tea and green tea (3.3, 3.2 and 2.3 c.u. respectively). Overall acceptability was based on the acceptability of general appearance, odour, consistency and taste and flavour. General appearance of control sample and the yoghurt with oolong tea and lemon balm (6.6, 6.5, 5.9 c.u.) were significantly more acceptable for the consumers than yoghurts with green and black tea (3.8 c.u., 4.3 c.u.). The most acceptable odour was that of yoghurt with oolong tea (7.0 c.u.) and control yoghurt (6.2 c.u.) while significantly less acceptable were the odours of yoghurts with green and black tea (4.4. c.u.) and with lemon balm (4.8 c.u.). Samples of yoghurts were also significantly different in taste acceptability which had big influence on the overall acceptability of those yoghurts. Yoghurt with oolong tea had the most acceptable taste (6.1 c.u.), control yoghurt has a significantly less acceptable taste (5.0 c.u.) and the least acceptable taste was that of yoghurt with black tea, yoghurt with lemon balm and with green tea (3.7 c.u., 3.3 c.u. and 2.6 c.u. respectively). Based on the consumer research, yoghurt with oolong tea had significantly the most acceptable consistency (6.3 c.u.), then yoghurt with green tea, black tea and lemon balm (4.4, 4.7 and 5.2 c.u.). It was found that three types of tea extract and lemon balm used in the yoghurt formula affected the acceptability and willingness to buy them by consumers. Of the plant additives used, green tea, oolong, black tea and lemon balm, the addition of oolong tea resulted in a higher acceptability of smell, consistency, but above all of taste, which resulted in a higher overall acceptability of oolong tea yoghurt compared to other plant additives and a comparable acceptability to control yoghurt, which also resulted in a greater willingness of consumers to buy oolong tea-infused yoghurt.

Product attributes that affected the level of acceptance were taste, ergonomics, overall form, cost of purchase and functional additives. On the other hand, low nutritional knowledge and lack of knowledge of production technology by the recipients might influence their willingness to try something that sounds foreign negatively, so products with the addition of unknown ingredients or with the use of modern technologies were not accepted by them [57]. The results indicated that yoghurts with innovative functional additives were highly valued by consumers, even better than a control product without additives, and would be willingly bought by them. During the food design process it is very important to use sensory methods to verify the quality of the developed products and their acceptability among consumers for whom the product is dedicated. In this paper the sensory quality of the developed yoghurts was verified by using expert methods such as Quantitative Descriptive Profile analyses and semi-consumer research where hedonic test were used. Both methods confirmed the possibility of using the tea additive, especially oolong teas, as an additive improving the quality of yoghurt by introducing into it a pleasant peach, nectar flavour and odour and at the same time its acceptability and the willingness to purchase it among a selected group of consumers. It should be further emphasised that studies conducted so far have mainly focused on the use of the addition of green tea, while our research indicates that semi-fermented oolong tea has a better effect on the sensory profile of yoghurt and its acceptance by consumers than green tea.

Some authors added different additives to the green tea yoghurt to mask their bitterness and increase their acceptability to consumers. Chatterjee and co-authors [21] developed green tea-infused yoghurts with 9% of chocolate syrup, 1% addition of skim milk powder and 3% of honey. All these ingredients were added to mask the bitterness of the green tea and enrich the taste of the product. Green tea-infused chocolate yoghurt’s overall acceptability was 7.35, while taste 6.64. Sugar or honey addition increased the overall acceptability (7.88 and 8.37 respectively) and taste (7.65 and 8.12 respectively). Yoghurt with chocolate syrup had a higher acceptability [21] than that assessed in these studies, which may have resulted from the addition of sugar syrup and honey to the product. In our yoghurt there were only simple ingredients necessary to create the yoghurt and to be in harmony with a clean label. We did not add sugar and any sugar substitutes or additives that increased the sweetness of the product, but we could observe from the sensory profile that the addition of oolong tea increased the perception of sweet taste in comparison with other yoghurt especially the control one. In the next study, it is worth considering the addition of a sweetener to tea yoghurt, but one that will not affect the nutritional value of the product, especially its caloricity, as this is the assumption of this research project. Up to now, studies that have been conducted on tea-infused yoghurt have only been evaluated using consumers [15,21,25,33]. Therefore, the extension of the research to include sensory evaluation by an expert panel allows us to obtain more information about the analyzed product and to verify the factors and attributes influencing its acceptability by consumers, as well as to provide guidelines for further work on the product formula and its possible reformulation.

### 3.2. Instrumental Analysis

The structure and the rheological properties of yoghurts are important to product quality and shelf life [42]. To evaluate the influence of different tea addition on the yoghurt texture, firmness and adhesiveness were tested. On the basis of statistical analysis, it was found that only the addition of oolong tea had an influence on the firmness of tested yogurts (Table 1). It was observed that oolong tea extract visibly reduces firmness and adhesiveness of yoghurt, which was positively marked in the acceptability of yoghurts and willingness to buy them by consumers in sensory analysis of the yoghurts. 

Other tea extract did not affect the structure of yoghurts, as their firmness and adhesiveness were very similar to the control sample, which was also confirmed by the sensory analysis. The literature data show that tea extract addition might even improve the texture of yogurts This phenomenon might be explained by the milk protein cross-linking with tea flavanols [35,58,59]. However, data in the literature points to the fact that the texture of tea-infused yogurts depends more on the quantity of added tea that on the kind of tea used [59].

Yield stress is an initial resistance of a probe to flow under stress and it is a measure of the interactions between the components in the product. For tested yoghurts, it was reported that yield stress (Table 1) was not generally influenced by the addition of tea; however it was reported that green tea influenced the yield stress by increasing it. The influence of green tea on the rheological properties of yoghurt such as apparent viscosity was also reported by Amirdivani and Baba [60]. This increase was probably caused by the presence of green tea polyphenolic compounds, which are able to interact with milk proteins [61].

**Figure 6:**
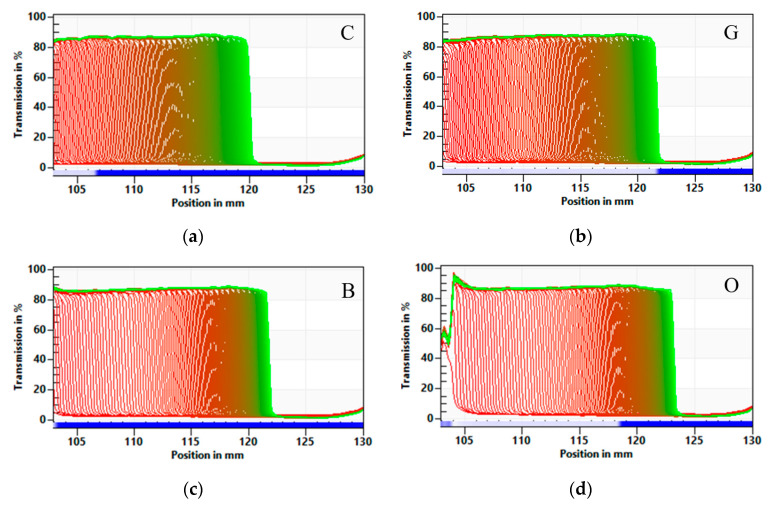

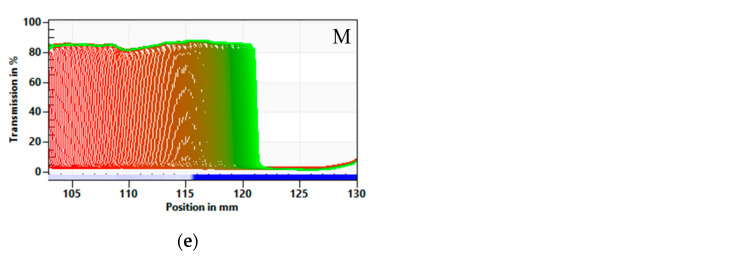
Influence of different tea infusion on the yoghurt transmission profiles presented enabling LUMiSizer^®^ analysis. (**a**) C—Control, (**b**) G—Green tea, (**c**) B—Black tea, (**d**) O—Oolong tea, (**e**) M—Lemon balm.

**Figure 7:**
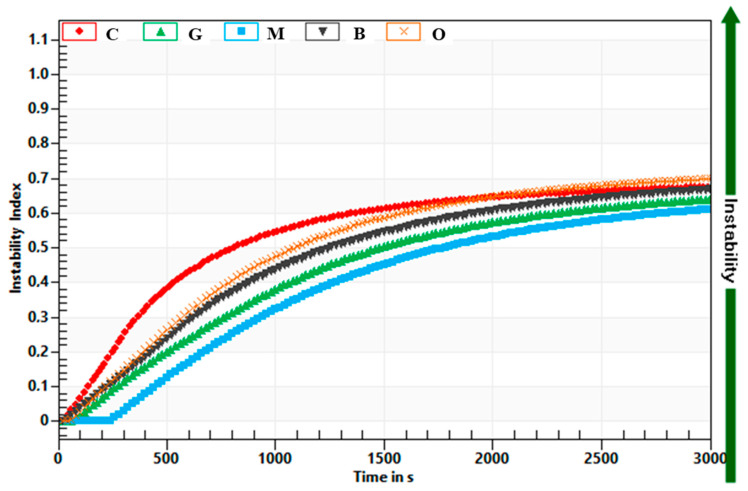
Influence of tea extract addition on stability of yogurt in comparison with plain yoghurt. C—Control, G—Green tea, B—Black tea, O—Oolong tea, M—Lemon balm.

The stability of yoghurts and possible syneresis, serum release from its structure, is regarded as a technological defect in yoghurts. That is why the characteristic of yoghurt stability is very important [62]. The effect of tea addition on yoghurts’ stability was examined with the multi-sample analytical centrifuge based on the STEP (space-time resolved extinction profiles) technology. Destabilisation progression of the process is shown in Figure 6. Addition of tea extracts did not affect the instability index (Table 1, Figure 7). All tested yoghurts were not stable during the time of stability determination. The serum release (syneresis), which was not influenced by tea extract addition, was observed. It was also observed in the plain yoghurt. The separation is known to be related to instability of yoghurt’s gel network and thus the loss in ability to entrap all the serum phase [63]. It is known from the literature data that polyphenols’ secondary plant metabolites presented in the tea extracts have the ability to interact with proteins, resulting in the formation of a protein–polyphenol complex, which are observed as weak interactions, mainly hydrophobic, van der Waals, hydrogen bridge-binding, and ionic interactions and might explain the lack of differences of stability of the tested yoghurts [62].

Colour is one of the most important visual attributes in yoghurts. The addition of tea to yoghurts significantly decreased the colour component value L* in comparison with the control sample (Table 2). During tea fermentation, an enzymatic oxidation of polyphenols, especially tea catechins, occurs. This leads to formation of a series of coloured chemical compounds, such as theaflavins (TFs) and thearubigins (TRs), which are responsible for the characteristics of the tea liquor’s colour and lightness which, in consequence, might influence the colour of the products obtained with tea [64]. The lowest L* values were noted for the yogurts with the green tea addition, whereas the brightest were yoghurts with lemon balm. The addition of tea also influenced the other colour parameters. Values of the a* component were the lowest for the control yoghurt as well as with green tea addition. The addition of other tested teas resulted in an increment of the a* parameter, which was due to natural colorants that were present in the tea. Also, the values of parameter b* varied between the control yogurts and yogurt with tea addition.

Among tested teas, the addition of black and oolong tea had a greater influence on the b* parameter. Those results were in correspondence with the data obtained by Liang et al. [64]. Based on comprehensive analysis of the effect of the tea addition on the colour of the yoghurts, the total colour difference parameter was calculated (ΔE). It was found that the differences in color between yoghurts with and without the addition of tea were noticeable even for the inexperienced observer (ΔE > 5) [46]. That colour analysis was confirmed by the Quantitative Descriptive Profile analysis where plain yoghurt was characterised by a light white colour while tea-infused yoghurts by a darker cream colour.

## 4. Conclusions

Among the analyzed green tea-, black tea-, oolong tea- and lemon balm-infused yoghurts, the addition of 2% oolong tea to the yoghurt allowed a functional food to be obtained with satisfactory texture, colour and sensory properties, accepted by consumers to the same degree as that of control yoghurt. The research conducted indicated that semi-fermented oolong tea had a better effect on the sensory profile of yoghurt evaluated by the expert panel and its acceptance by consumers than green tea, and these studies can be used to commercialise oolong tea-infused yoghurt. However, further research is planned to increase the acceptability of the product by adding other natural additives which can increase the sensory quality and acceptability of the product, as well as storage research.

## Figures and Tables

**Table 1:** Physical properties of tea infused yoghurts. C—Control, G—Green tea, B—Black tea, O—Oolong tea, M—Lemon balm.

Type of Tea	Firmness [N]	Adhesiveness [Ns]	Yield Stress [Pa]	Instability Index
C	0.73 ^b^ ± 0.01	−1.75 ^a^ ± 0.03	130.67 ^ab^ ± 3.00	0.674 ^a^ ± 0.008
G	0.84 ^b^ ± 0.03	−2.23 ^a^ ± 0.05	175.43 ^b^ ± 2.83	0.642 ^a^ ± 0.086
B	0.69 ^ab^ ± 0.07	−1.51 ^ab^ ± 0.05	121.20 ^ab^ ± 2.91	0.686 ^a^ ± 0.011
O	0.43 ^a^ ± 0.02	−1.00 ^b^ ± 0.06	103.83 ^a^ ± 2.65	0.698 ^a^ ± 0.010
M	0.68 ^ab^ ± 0.03	−1.65 ^a^ ± 0.07	112.13 ^a^ ± 0.07	0.615 ^a^ ± 0.062

Values are mean ± SD (*n* = 3), ^a–d^—values followed by the same letter within a column do not differ significantly according to Tukey’s test (*p* < 0,05).

**Table 2:** Colour parameters and the total colour difference parameter of yogurts obtained without or with the addition of tea. C—Control, G—Green tea, B—Black tea, O—Oolong tea, M—Lemon balm.

Type of Tea	Colour Parameters			ΔE ^#^
	L*	a*	b*	
C	90.22 ^d^ ± 0.22	−0.83 ^a^ ± 0.04	10.29 ^a^ ± 0.12	
G	77.98 ^a^ ± 0.06	−0.88 ^a^ ± 0.09	12.52 ^b^ ± 0.28	12.45 ± 0.15
B	81.76 ^b^ ± 0.22	2.78 ^d^ ± 0.12	17.52 ^d^ ± 0.15	11.71 ± 0.43
O	81.85 ^b^ ± 1.52	1.07 ^c^ ± 0.18	16.86 ^d^ ± 0.52	10.84 ± 2.06
M	87.45 ^c^ ± 0.17	0.66 ^b^ ± 0.06	14.49 ^c^ ± 0.21	5.26 ± 0.08

Values are mean ± SD (*n* = 3). ^a, b^—values followed by the same letter within a column do not differ significantly according to Tukey’s test (α = 0.05). ^#^ total colour difference parameter calculated in relation to the control sample.

## Supplementary Materials

The following are available online at https://www.mdpi.com/2304-8158/9/12/1848/s1, Table S1: Sensory attribute for yoghurts profiling, their definitions and intensity range, Table S2: Sensory characteristics of yoghurts evaluated by experts: C—Control, G—Green tea, B—Black tea, O—Oolong tea, M—Lemon balm- the mean values, 2 sessions (*n* = 20), Table S3: Acceptability of yoghurts and willingness to buy evaluated by consumers: C—Control, G—Green tea, B—Black tea, O—Oolong tea, M—Lemon balm- the mean values (*n* = 30).
