# Supplementary material for: Functional Tea-Infused Set Yoghurt Development by Evaluation of Sensory Quality and Textural Properties
Source: Foods. 2020 Dec 11;9(12):1848. doi: 10.3390/foods9121848 (PMC7763908; doi:10.3390/foods9121848)
Supplement: Supplementary file 1 [file foods-09-01848-s001.pdf]

Supplementary Materials

Table 1. Sensory attribute for yoghurts profiling, their definitions and intensity range.

Attributes	Definitions	Intensity range
	**Odour**	
**Sweet**	Basic odour quality - no definition required	none - very intensive
**Sour**	Basic odour quality - no definition required	none - very intensive
**Yoghurt**	Basic odour quality - no definition required	none - very intensive
**Milky**	Odour of milk	none - very intensive
**Fat**	Odour of fat milk	none - very intensive
**Green tea**	Odour characteristic for the green tea	none - very intensive
**Black tea**	Odour characteristic for the black tea	none - very intensive
**Herbs**	Odour characteristic for the herbs	none - very intensive
**Peach**	Odour characteristic for the peach fruit	none - very intensive
**Citrus**	Odour characteristic for the citrus fruits	none - very intensive
**Nectar**	Odour characteristic for the nectar and flowers	none - very intensive
	**Visual attributes**	
**Whey**	Presence and amount of whey in the product's surface	none - a lot of
**Shine**	Shine of the surface	mat - shiny
**Colour**	Colour intensity of the product	light white - dark cream
**Smoothness**	Degree of smoothness in the product's surface (grainy/ smooth appearance)	rough - smooth
**Adhesiveness**	Perception of the product's density measured by the resistance of the spoon	thin - thick
**Filling the teaspoon**	Way to fill a teaspoon	flat - conical
**Consistency uniformity**	Perceived visually uniformity of the product	not uniform - uniform
	**Texture in mouth**	
**Thickness**	Perception of the product's density in the mouth	thin - thick
**Melting**	Degree of the product's melting in the mouth	much delayed - immediate
**Firmness**	The degree of firmness of the product's consistency	loose – firm
**Yield stress**	The degree of viscosity of the sample	low viscosity- high viscosity
**Fat film**	Perception of the fatty film, coating the oral cavity with the fatty film	none - very intensive
**Creaminess**	Impression of creaminess connected with melting yoghurt in the mouth, in contrast to thin sample	thin - creamy
**Smoothness**	Perception of smoothness in the mouth	rough - smooth
	**Basic taste**	
**Acid**	Basic taste quality - no definition required	none - very intensive
**Sweet**	Basic taste quality - no definition required	none - very intensive
**Bitter**	Basic taste quality - no definition required	none - very intensive
**Astringent**	Perception of astringency in the mouth	none - very intensive
	**Flavour**	
**Milky**	Flavour of milk	none - very intensive
**Yoghurt**	Flavour of yoghurt	none - very intensive
**Quark**	Flavour of quark (fresh cheese)	none - very intensive
**Green tea**	Flavour of infused green tea	none - very intensive
**Black tea**	Flavour of infused black tea	none - very intensive
**Herbs**	Flavour of infused melissa	none - very intensive
**Peach**	Flavour of peach fruit	none - very intensive
**Nectar**	Flavour of nectar and flowers	none - very intensive

**Body**	Harmonization of all positive attributes	incomplete – full
**Overall quality**	Overall impression of the harmony of positive attributes, with no or only slight negative notes	low–high

Table 2. Sensory characteristics of yoghurts evaluated by experts: C – Control, G – Green tea, B – Black tea, O – Oolong tea, M – Lemon balm- the mean values, 2 sessions (n=20).

		**C**	**G**	**B**	**O**	**M**
	o. milky	4.0^{c}	3.5^{bc}	3.4^{bc}	2.8^{ab}	2.0^{a}
	o. yoghurt	4.7^{c}	4.0^{bc}	3.3^{b}	3.4^{b}	2.1^{a}
	o. sour	4.0^{b}	2.8^{a}	2.7^{a}	2.6^{a}	2.2^{a}
	o. sweet	1.5^{a}	2.2^{ab}	2.7^{b}	2.9^{b}	1.5^{a}
	o. fat	3.0^{b}	3.1^{b}	2.6^{ab}	2.6^{ab}	1.7^{a}
**odour**	o. green tea	0.0^{a}	2.5^{c}	0.2^{a}	0.5^{a}	1.6^{b}
	o. black tea	0.0^{a}	0.5^{a}	2.1^{b}	0.1^{a}	0.2^{a}
	o. herbs	0.0^{a}	0.6^{ab}	1.0^{b}	0.1^{a}	4.1^{c}
	o. peach	0.0^{a}	2.1^{bc}	3.1^{c}	4.4^{d}	1.3^{b}
	o. citrus	0.0^{a}	0.9^{b}	0.8^{b}	1.4^{b}	1.0^{b}
	o. nectar	0.0^{a}	1.5^{b}	1.7^{b}	2.1^{b}	1.3^{b}
	whey	1.9^{a}	4.8^{b}	4.7^{b}	4.9^{b}	4.5^{b}
	shine	6.8	7.3	7.2	7.5	7.4
	colour intensity	1.0^{a}	4.3^{c}	5.2^{cd}	3.2^{b}	5.8^{d}
**appearance**	visually smoothness	6.8	7.0	6.4	7.1	6.1
	adhesiveness	6.0	6.2	6.6	6.9	6.5
	filling the teaspoon	6.0	6.5	6.1	6.8	6.6
	uniformity of consistency	6.4	5.2	5.3	6.4	5.6
	thickness in the mouth	5.3	5.6	5.1	5.1	5.4
	melting	6.7	6.5	6.4	6.4	7.2
	firmness	5.3	5.3	5.4	5.8	5.6
**texture in the mouth**	yield stress	2.1	2.7	2.7	2.5	2.4
	fat film	2.0	2.3	2.0	2.2	2.1
	creaminess	4.2	4.4	3.9	5.2	4.7
	smoothness in the mouth	6.7	6.2	6.2	6.8	6.7
	t. sour	5.8	5.0	5.1	4.9	4.5
	t. sweet	1.0	1.1	1.2	1.7	0.9
	t. bitter	0.8	2.1	1.7	1.5	2.0
	t. astringent	2.0	2.1	2.3	2.5	2.2
	f. milky	3.3^{b}	1.9^{a}	1.9^{a}	2.1^{a}	1.6^{a}
**taste /flavour**	f. yoghurt	5.1^{b}	3.3^{a}	3.0^{a}	3.3^{a}	2.8^{a}
	f. quark	2.5	1.5	2.2	1.4	1.3
	f. green tea	0.0^{a}	3.5^{d}	1.3^{ab}	0.9^{b}	2.3^{c}
	f. black tea	0.0^{a}	0.4^{a}	1.7^{b}	0.1^{a}	0.3^{a}
	f. herbs	0.0^{a}	0.9^{b}	0.9^{b}	0.5^{ab}	4.6^{c}
	f. peach	0.0^{a}	1.4^{b}	1.7^{b}	3.8^{c}	0.9^{b}
	f. nectar	0.0^{a}	1.0^{b}	0.7^{b}	1.5^{b}	0.9^{b}
**body**	body	5.1^{c}	3.8^{a}	3.9^{ab}	5.0^{bc}	3.9^{ab}
**quality**	overall quality	5.9^{c}	4.5^{ab}	4.4^{ab}	5.6^{bc}	4.0^{a}

Mean values marked with different indices a, b, c, d differ statistically ($p \leq 0.05$).

Table 3. Acceptability of yoghurts and willingness to buy evaluated by consumers: C – Control, G – Green tea, B – Black tea, O – Oolong tea, M – Lemon balm- the mean values (n=30).

	C	G	B	O	M
appearance	6.6^{b}	3.8^{a}	4.3^{a}	6.5^{b}	5.9^{b}
odour	6.2^{b}	4.4^{a}	4.4^{a}	7.0^{b}	4.8^{a}
consistency	5.9^{bc}	4.4^{a}	4.7^{a}	6.3^{c}	5.2^{ab}
flavour	5.0^{c}	2.6^{a}	3.7^{b}	6.1^{d}	3.3^{ab}
overall acceptability	5.7^{b}	3.0^{a}	3.7^{a}	5.9^{b}	3.8^{a}
willingness to buy	5.3^{b}	2.3^{a}	3.2^{a}	5.6^{b}	3.3^{a}

Mean values marked with different indices a, b, c, d differ statistically ($p \leq 0.05$).
